# Association of commuting mode with dyslipidemia and its components after accounting for air pollution in the working population of Beijing, China

**DOI:** 10.1186/s12889-019-6887-x

**Published:** 2019-05-22

**Authors:** Lixin Tao, Xia Li, Jie Zhang, Jia Liu, Yue Liu, Haibin Li, Xiangtong Liu, Yanxia Luo, Xiuhua Guo

**Affiliations:** 1 School of Public Health, Capital Medical University, Beijing, China; 2 Beijing Municipal Key Laboratory of Clinical Epidemiology, Beijing, China; 3 Department of Mathematics and Statistics, La Trobe University, Melbourne, Australia

**Keywords:** Dyslipidemia, Commuting mode, Air pollution, Working population

## Abstract

**Background:**

Evidence of the association between dyslipidemia and its components with commuting mode after adjusting for air pollution is limited. This study aimed to explore the association of dyslipidemia and its components with the modes used to commute to and from work after accounting for air pollution and other potential confounding factors.

**Methods:**

This cross–sectional study was based on data collected from a working population of 69 functional communities in Beijing in 2016. A final sample of 8090 adults aged 18–65 years (mean age: 38.36 ± 9.75 years) was enrolled in the study. Risk estimates and their corresponding 95% confidence intervals (CIs) for the associations of dyslipidemia and its components with commuting mode were determined using multivariate logistic regression models.

**Results:**

Of the 8090 subjects, 2419 (29.90%) met the criteria for dyslipidemia. Compared with car or taxi commuters, walking (OR 0.79, 95% CI 0.64 to 0.97), cycling (OR 0.71, 95% CI 0.58 to 0.86) and bus-riding commuters (OR 0.78, 95% CI 0.66 to 0.91) had a lower risk for dyslipidemia. Compared with car or taxi commuting, walking, cycling and bus-riding commuting were also associated with a lower risk for some components of dyslipidemia. Among the walking, cycling and bus-riding commuters, a dose-response trend of the association between dyslipidemia, some of its components and commuting mode by commuting time was also observed.

**Conclusions:**

Walking, cycling and bus-riding commuting can reduce the risk for dyslipidemia and some of its components. Education on the prevention of dyslipidemia should be emphasized among higher-risk people who usually commute by car or taxi. Population-wide health may be improved by policies that encourage active commuting, particularly cycling and walking.

**Electronic supplementary material:**

The online version of this article (10.1186/s12889-019-6887-x) contains supplementary material, which is available to authorized users.

## Background

While the prevalence of dyslipidemia has increased [1], there has been a decrease in the level of physical activity and an increase in sedentary time [2, 3]. Physical inactivity increases the risk for developing many chronic diseases, such as type 2 diabetes [4, 5], cardiovascular diseases (CVDs) [6, 7] and some cancers [8]. Active commuting is defined as utilizing a human-powered transportation mode, such as walking, cycling or other active commuting methods. One previous study on the working population demonstrated that before the year 2000, active commuting modes such as walking and cycling were the main commuting mode, but today an increasing number of people have cars and drive to work in Beijing [9]. It is suggested that Beijing is one of the cities with the highest rate of car owner-ship [10]. Furthermore, the number of vehicles in Beijing is expected to be 10.4 million by 2030 [11]. Because of this rapid increase, cars and taxis have become some of the dominant modes of travel in Beijing. Active commuting to work has been recommended in light of the increasing prevalence of sedentary lifestyles as a promising approach to increase daily physical activity. Epidemiological evidence has proven that regular active commuting is associated with beneficial effects for a range of health outcomes [12], such as chronic diseases [13] and mortality [14] in children and adults. A prospective cohort study reported that commuting via cycling was associated with a reduction in the risk of incident CVD, cancer and all-cause mortality [15]. Walking commuting was also considered to be associated with a lower risk of CVD [15]. A meta–analysis including 531,333 participants also reported that active commuting by walking or cycling reduces the risk for CVD [12]. A dose-response meta-analysis on the relationship between walking and cycling and all-cause mortality demonstrated a significantly reduced risk for all-cause mortality, with the greatest effects observed for 120 min per week of walking and 100 min per week of cycling in the adult population [14].

A number of studies have demonstrated that high levels of total cholesterol (TC) and low-density lipoprotein cholesterol (LDL-C) are the major risk factors for CVD [16, 17]. It was also suggested that decreased high-density lipoprotein cholesterol (HDL-C) levels can increase the risk for CVD [18]. Evidence from epidemiologic, genetic, and biological studies strongly indicates that elevated triglycerides (TGs) represent a causal risk factor for CVD [19]. Active commuting can be an appealing way to facilitate daily, routine physical activity and may have protective effects against dyslipidemia [9] and CVD [12, 13]. One existing study explored the association between commuting mode and dyslipidemia [9]. The study showed that people who go to work by bus, car or taxi have a higher risk for dyslipidemia than those who walk to work. No significant difference was found in the risk of dyslipidemia between workers who ride bicycles and with those who walk to work [9]. Previous studies have also found a negative association between active commuting and other chronic diseases and indices, such as CVD [19, 20], metabolic syndrome [21], obesity [22–24], body mass index (BMI) [25–29], elevated blood pressure [30] and mental health [31]. In these studies, the researchers adjusted for dietary behavior [23, 24], physical activity [21–27, 30, 31] and many other potential confounders, such as demographic factors, including age, gender, educational level, and lifestyle factors, including alcohol intake and smoking status. However, most studies on the association between commuting mode and health outcomes did not take the effect of air pollution into account. To our knowledge, only one cross-sectional study assessing the relationship between cycle commuting and perceived stress adjusted for environmental factors [32]. Exposure to ambient air pollution increases mortality and morbidity and is a leading cause of the global disease burden [33]. The Global Burden of Disease 2015 study revealed that approximately 1.1 million people died prematurely and 21.8 million disability-adjusted life-years were lost because of ambient air pollution in China in 2015 [33]. In 2017, it was estimated that health loss associated with PM_2.5_ in 74 leading cites in China included 365,400 premature deaths attributable to PM_2.5_ pollution, which comprised 50,470 cases of COPD, 97,790 cases of ischaemic heart disease, 43,000 cases of lung cancer, and 174,140 cases of stroke [34]. Epidemiological evidence has indicated that air pollutants may disrupt cholesterol metabolism [35], increase the levels of TC [36], TGs [37], and apolipoprotein-B [35] and reduce the HDL-C level [35]. Traffic-related air pollution have adverse effects on human health. It is vital to adjust for the effects of air pollution when evaluating the association of dyslipidemia and its components with commuting mode. To our knowledge, there are no existing studies examining the relationship of dyslipidemia and its components with commuting mode after adjusting for environmental factors. Consequently, there is a need to understand the relationship between commuting mode and dyslipidemia after taking air pollution into account.

Therefore, the aim of this study was to identify the modes of commuting to and from work in Beijing and investigate the association of dyslipidemia and its components (elevated TC, elevated TGs, reduced HDL-C, elevated LDL-C and elevated non-HDL-C) with commuting mode after adjusting for air pollution as well as other potential confounding factors.

## Methods

### Study design and participants

The cross-sectional study was based on data collected from a working population of 69 functional communities by performing health check-ups at the Beijing Physical Examination Center and Beijing Xiaotangshan Hospital from 1 January to 31 December 2016. From an initial convenience sample, 8332 adults who attended health check-ups and who provided information about their commuting mode were enrolled in this study. Of the total 8332 subjects, 67 subjects who were unemployed or retired and 175 subjects with a previous diagnosis of CVD, cerebrovascular disease or cancer were excluded from the study, leaving 8090 subjects (mean age: 38.36 ± 9.75 years; age range: 18–65 years; 56.13% males) in the present study (97.10% response rate).

### Data collection

The participants underwent health check-ups that included the measurement of height, weight, and blood pressure; overnight fasting blood sampling; and a questionnaire. Anthropometric measurements were performed with the participant lightly clothed and standing without shoes. Blood pressure was measured for each subject in a sitting position after at least a 5-min resting period. The subjects were required to refrain from smoking or consuming caffeine in the 30-min preceding the measurements. Three readings each of systolic and diastolic blood pressures were recorded, with an interval of at least 1 min, and the average of the last two measurements was used for data analysis. Mean arterial pressure (MAP) was calculated as diastolic blood pressure (DBP) plus one third of the difference in systolic blood pressure (SBP) minus the DBP.

Blood samples were collected from subjects after an overnight fast of at least 12 h. TC, TGs, HDL–C, LDL-C and fasting plasma glucose (FPG) were measured by an enzymatic method using a chemistry analyzer (Beckman LX 20, America) at the central laboratory of the hospitals. All analyses were performed in accordance with the manufacturer’s recommendations.

Sociodemographic and lifestyle data were obtained through a self-reported questionnaire; the data obtained were as follows: age (< 45 years old, ≥ 45 years old), gender (male, female), BMI (< 28 kg/m^2^, ≥ 28 kg/m^2^), education level (high school or lower, college, graduate degree or above), self-reported work stress (low, moderate, high), physical activity frequency (less than once every week, more than once every week, more than once every day), physical activity intensity [mild (walking, Tai Chi, dancing), moderate (jogging, cycling, climbing), vigorous (swimming, playing ball games, rope skipping)], sleep duration (< 6 h/day, ≥ 6 h/day), smoking status (yes, no), alcohol consumption status (yes, no), vegetable intake (yes, no), excessive meat intake (yes, no), excessive fat intake (yes, no), excessive salt intake (yes, no), excessive sugar intake (yes, no), and medication history for hypertension (yes, no), diabetes (yes, no), and dyslipidemia (yes, no). Information concerning commuting mode was based on the following two questions: “How do you normally travel to and from work? (driving a car or taking a taxi, walking, cycling, taking a bus, taking the subway)” and “How much time do you spend travelling to or from work in a typical day?”

The air pollution data, including PM_2.5_, PM_10_, NO_2_, SO_2_, CO and O_3,_ were obtained from the Centre of City Environmental Protection Monitoring Website Platform (www.bjmemc.com.cn) based on the period from January 1, 2015 to December 31, 2016 in Beijing. There were 35 monitoring stations located in 16 districts of Beijing. The daily mean concentration of air pollutants in each district was calculated by averaging the concentrations of all stations in that district. To account for the long-term effect of air pollution on dyslipidemia, one-year average concentrations of air pollutants before the date of the health check-ups for subjects in 16 districts of Beijing were used.

### Measurements

Dyslipidemia was diagnosed if the subjects had one or more abnormal serum lipid concentration or used antidyslipidemic medications, according to the 2016 Chinese guidelines for the management of dyslipidemia in adults [38]. The cut-off values for elevated TC, elevated TGs, reduced HDL-C, elevated LDL-C and elevated non-HDL-C were as follows:

1. Elevated TC ≥ 6.2 mmol/L (240 mg/dl);
2. Elevated TGs ≥ 2.3 mmol/L (200 mg/dL);
3. Reduced HDL-C < 1.0 mmol/L (40 mg/dL);
4. Elevated LDL-C ≥ 4.1 mmol/L (160 mg/dL); and
5. Elevated non-HDL-C ≥ 4.9 mmol/L (190 mg/dL). Non-HDL-C was calculated by subtracting HDL-C from TC.

### Statistical analysis

Continuous variables are presented as the means and standard deviations for normally distributed data or medians and interquartile ranges (25–75%) for data with a skewed distribution, and categorical variables are expressed as numbers and percentages. The characteristics of the participants were compared among the five different commuting mode groups (driving a car or taking a taxi, walking, cycling, taking a bus, taking the subway). The Kruskal–Wallis rank test was used among the five commuting groups for continuous variables with a skewed distribution. Chi–square tests were used for nominal categorical variables, and Kruskal–Wallis rank tests were used for ordinal categorical variables among the five commuting groups (driving a car or taking a taxi, walking, cycling, taking a bus, taking the subway). Multivariate logistic regression models were performed to estimate the odds ratios (ORs) and 95% confidence intervals (CIs) of the commuting modes for dyslipidemia and its components (elevated TC, elevated TGs, reduced HDL-C, elevated LDL-C and elevated non-HDL-C). The dose-response trend of the association of dyslipidemia and its components with commuting mode by commuting time was also explored. For the walking or cycling commuting modes, less than 30-min was categorized as a short time and longer than 30-min was categorized as a long time. For the bus commuting mode, less than 30-min was categorized as a short time, longer than 30-min and shorter than 60-min was categorized as a moderate time, and longer than 60-min was categorized as a long time. The mode of commuting was included in the models as the dependent variable (reference: car or taxi commuting mode). In these models, the PM_2.5_, PM_10_, NO_2_, SO_2_, CO and O_3_ concentrations used were one-year averages from before the date of the health check-ups for every subject in 16 districts of Beijing.

## Results

### Sociodemographic, clinical and behavioral factors of study participants

**Fig. 1:**
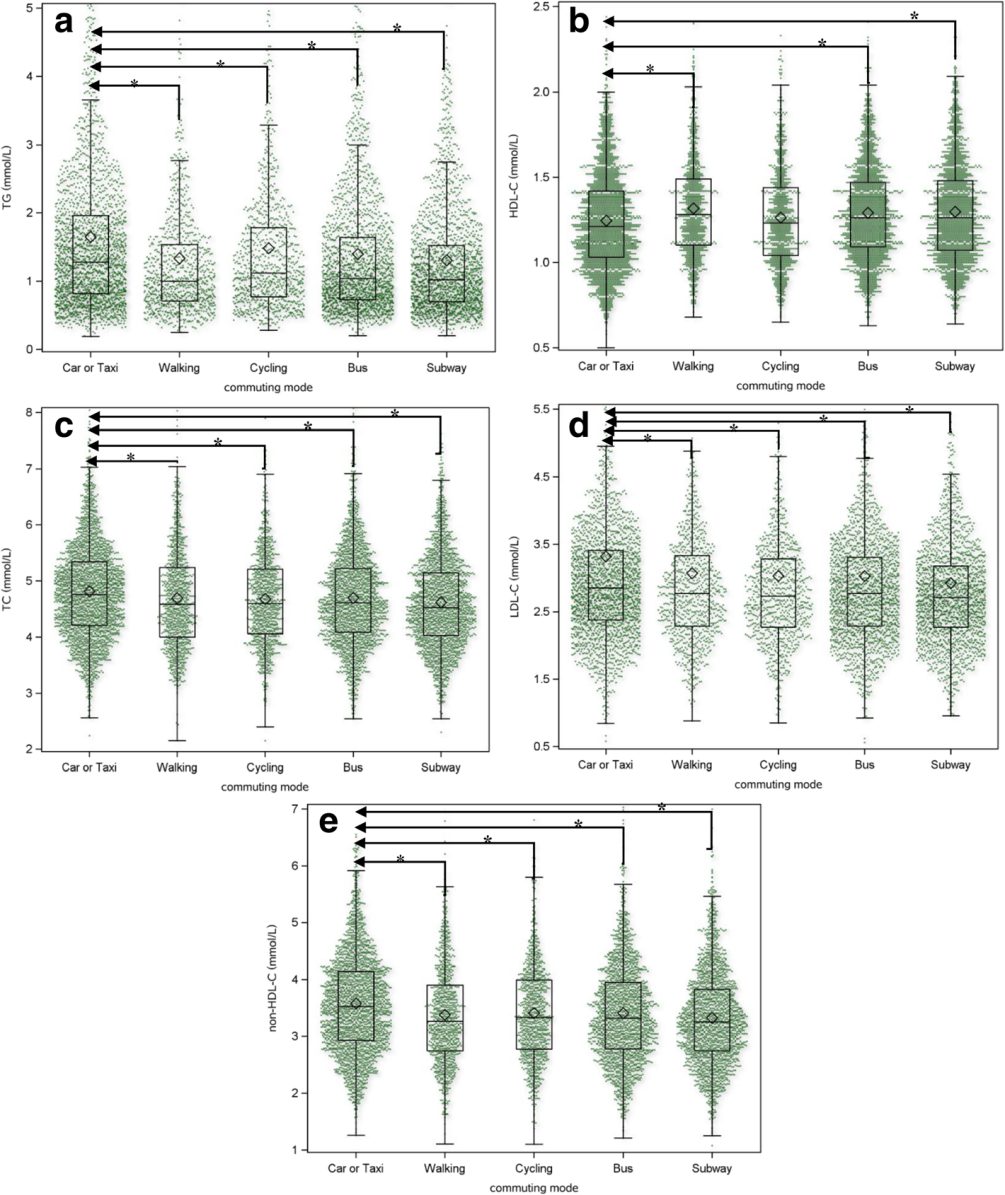
The levels of five components of dyslipidemia by commuting mode. *TGs,* triglycerides; *HDL-C,* high-density lipoprotein cholesterol; *TC,* total cholesterol; *LDL-C,* low-density lipoprotein cholesterol; *non-HDL-C,* non-high-density lipoprotein cholesterol. *: *p* < 0.05

The general characteristics of the study participants by commuting category are summarized in Table 1, Table 2 and Fig. 1. The prevalence of dyslipidemia, elevated TGs, reduced HDL-C, elevated TC, elevated LDL-C and elevated non-HDL-C are shown in Table 1. Of the 8090 subjects, 2419 (29.90%) met the criteria for dyslipidemia, 426 (5.27%) met the criteria for elevated TC, 1120 (13.84%) met the criteria for elevated TGs, 595 (7.35%) met the criteria for elevated LDL-C, 1481 (18.31%) met the criteria for reduced HDL-C, and 441 (5.45%) met the criteria for elevated non-HDL-C. Figure 1 shows that car or taxi commuters had the highest levels of TGs, TC, LDL-C, and non-HDL-C and the lowest HDL-C levels among the commuters in the five commuting mode groups. There were significant differences between car or taxi commuters and the individuals in the other four commuting mode groups in TGs, TC, LDL-C, HDL-C, and non-HDL-C levels. No significant difference was found between car or taxi commuters and cycling commuters in HDL-C.

**Table 1:** Sociodemographic and clinical characteristics of the study subjects by commuting mode

Characteristics	Total (*n* = 8090)	Car or Taxi (*n* = 2325)	Walking (*n* = 957)	Cycling (*n* = 933)	Bus (*n* = 1937)	Subway (*n* = 1938)	*P* value
Gender (male), *n* (%)	4541 (56.13)	1452 (31.98)	512 (11.28)	610 (13.43)	1003 (22.09)	964 (21.23)	< 0.0001
Age (years)	37 (31–46)	39.00 (33.00–47.00)	36.00 (28.00–47.00)	40.00 (33.00–48.00)	37.00 (30.00–47.00)	34.00 (29.00–40.00)	< 0.0001
BMI (kg/m^2^)	23.9 (21.40–26.40)	24.40 (22.00–27.10)	23.50 (21.20–26.00)	24.50 (22.00–26.80)	23.60 (21.20–26.00)	23.40 (21.10–26.00)	< 0.0001
MAP (mmHg)	93.33 (83.33–100.00)	93.33 (86.67–100.67)	93.33 (83.33–100.00)	93.33 (90.00–103.33)	93.33 (84.00–100.00)	90.00 (83.33–96.67)	< 0.0001
FPG (mmol/L)	5.13 (4.83–5.49)	5.18 (4.87–5.54)	5.11 (4.81–5.49)	5.18 (4.89–5.55)	5.12 (4.81–5.49)	5.07 (4.79–5.39)	< 0.0001
TC (mmol/L)	4.63 (4.09–5.23)	4.76 (4.21–5.34)	4.59 (4.00–5.24)	4.60 (4.06–5.21)	4.61 (4.09–5.22)	4.53 (4.03–5.14)	< 0.0001
TGs (mmol/L)	1.10 (0.74–1.71)	1.28 (0.82–1.96)	1.00 (0.71–1.54)	1.12 (0.77–1.78)	1.04 (0.73–1.64)	1.02 (0.70–1.52)	< 0.0001
HDL-C (mmol/L)	1.24 (1.06–1.46)	1.21 (1.03–1.42)	1.28 (1.10–1.49)	1.23 (1.04–1.44)	1.26 (1.09–1.47)	1.26 (1.07–1.48)	< 0.0001
LDL-C (mmol/L)	2.77 (2.31–3.31)	2.85 (2.38–3.41)	2.77 (2.29–3.33)	2.73 (2.27–3.28)	2.77 (2.29–3.31)	2.71 (2.27–3.18)	< 0.0001
Non-HDL-C (mmol/L)	3.35 (2.80–3.98)	3.52 (2.93–4.14)	3.27 (2.74–3.90)	3.33 (2.77–3.99)	3.32 (2.78–3.95)	3.25 (2.74–3.83)	< 0.0001
Commuting time (hours)	1.00 (0.50–1.00)	1.00 (0.50–1.00)	0.50 (0.20–0.50)	0.50 (0.50–1.00)	1.00 (1.00–1.50)	1.00 (1.00–1.50)	< 0.0001
Dyslipidemia (*n*, %)	2419 (29.90)	836 (35.96)	272 (28.42)	275 (29.47)	520 (26.85)	516 (26.63)	< 0.0001
Elevated TC (*n*, %)	426 (5.27)	148 (6.37)	59 (6.17)	42 (4.50)	93 (4.80)	84 (4.33)	0.0141
Elevated TGs (*n*, %)	1120 (13.84)	432 (18.58)	102 (10.66)	126 (13.50)	250 (12.91)	210 (10.84)	< 0.0001
Elevated LDL-C (*n*, %)	595 (7.35)	220 (9.46)	74 (7.73)	62 (6.65)	126 (6.50)	113 (5.83)	0.0001
Reduced HDL-C (*n*, %)	1481 (18.31)	528 (22.71)	158 (16.51)	172 (18.44)	301 (15.54)	322 (16.62)	0.0001
Elevated non-HDL-C (*n*, %)	441 (5.45)	166 (7.14)	53 (5.54)	41 (4.39)	97 (5.01)	84 (4.33)	0.0004
Age group (*n*, %)							< 0.0001
< 45 years old	5812 (71.84)	1577 (67.83)	665 (69.49)	623 (66.77)	1297 (66.96)	1650 (85.14)	
≥ 45 years old	2278 (28.16)	748 (32.17)	292 (30.51)	310 (33.23)	640 (33.04)	288 (14.86)	
BMI group							< 0.0001
< 28 kg/m^2^	6997 (86.49)	1948 (83.78)	840 (87.77)	790 (84.67)	1702 (87.87)	1717 (88.60)	
≥ 28 kg/m^2^	1093 (13.51)	377 (16.22)	117 (12.23)	143 (15.33)	235 (12.13)	221 (11.40)	
Education level (*n*, %)							< 0.0001
High school or lower	963 (11.90)	177 (7.61)	185 (19.33)	212 (22.72)	271 (13.99)	118 (6.09)	
College	5228 (64.62)	1652 (71.05)	572 (59.77)	537 (57.56)	1232 (63.60)	1235 (63.73)	
Graduate degree or above	1899 (23.47)	496 (21.33)	200 (20.90)	184 (19.72)	434 (22.41)	585 (30.19)	

Data are presented as the median (interquartile range) or n (%)

Abbreviations: *BMI* body mass index, *MAP* mean arterial pressure, *FPG* fasting plasma glucose, *TC* total cholesterol, *TGs* triglycerides, *LDL-C* low-density lipoprotein cholesterol, *HDL-C* high-density lipoprotein cholesterol, *non-HDL-C* non-high-density lipoprotein cholesterol

**Table 2:** Behavioral factors of the study subjects by commuting mode

Characteristics	Total (*n* = 8090)	Car or Taxi (*n* = 2325)	Walking (*n* = 957)	Cycling (*n* = 933)	Bus (*n* = 1937)	Subway (*n* = 1938)	*P* value
Self–reported work stress (*n*, %)							< 0.0001
Low	1155 (14.28)	292 (12.56)	154 (16.09)	155 (16.61)	323 (16.68)	231 (11.92)	
Moderate	3580 (44.25)	989 (42.54)	445 (46.50)	398 (42.66)	840 (43.37)	908 (46.85)	
High	3355 (41.47)	1044 (44.90)	358 (37.41)	380 (40.73)	774 (39.96)	799 (41.23)	
Physical activity frequency (*n*, %)							< 0.0001
Less than once every week	4078 (50.41)	1158 (49.81)	346 (36.15)	417 (44.69)	1065 (54.98)	1092 (56.35)	
More than once every week	2545 (31.46)	793 (34.11)	301 (31.45)	285 (30.55)	558 (28.81)	608 (31.37)	
More than once every day	1467 (18.13)	374 (16.09)	310 (32.39)	231 (24.76)	314 (16.21)	238 (12.28)	
Physical activity intensity (*n*, %)							< 0.0001
Mild (walking, Tai Chi, dancing)	5525 (68.29)	1579 (67.91)	596 (62.28)	549 (58.84)	1408 (72.69)	1393 (71.88)	
Moderate (jogging, cycling, climbing)	1869 (23.10)	500 (21.51)	252 (26.33)	300 (32.15)	413 (21.32)	404 (20.85)	
Vigorous (swimming, playing ball games, rope skipping)	696 (8.60)	246 (10.58)	109 (11.39)	84 (9.00)	116 (5.99)	141 (7.28)	
Sleep duration (*n*, %)							< 0.0001
< 6 h/day	7212 (89.15)	2036 (87.57)	843 (88.09)	842 (90.25)	1708 (88.18)	1783 (92.00)	
≥ 6 h/day	878 (10.85)	289 (12.43)	114 (11.91)	91 (9.75)	229 (11.82)	155 (8.00)	
Current or previous smoker (*n*, %)	1957 (24.19)	766 (32.95)	211 (22.05)	254 (27.22)	381 (19.67)	345 (17.80)	< 0.0001
Current or previous alcohol consumption (*n*, %)	3240 (40.05)	1076 (46.28)	371 (38.77)	404 (43.30)	706 (36.45)	683 (35.24)	< 0.0001
High vegetable intake (n, %)	1014 (12.53)	265 (11.40)	127 (13.27)	108 (11.58)	249 (12.85)	265 (13.67)	0.1666
Excessive meat intake (*n*, %)	1086 (13.42)	389 (16.73)	102 (10.66)	118 (12.65)	220 (11.36)	257 (13.26)	< 0.0001
Excessive fat intake (*n*, %)	387 (4.78)	91 (3.91)	52 (5.43)	38 (4.07)	89 (4.59)	117 (6.04)	0.0133
Excessive salt intake (*n*, %)	1746 (21.58)	522 (22.45)	202 (21.11)	218 (23.37)	430 (22.20)	374 (19.30)	0.0534
Excessive sugar intake (*n*, %)	722 (8.92)	209 (8.99)	72 (7.52)	79 (8.47)	186 (9.60)	176 (9.08)	0.4456
Medication history for hypertension (*n*, %)	593 (7.33)	206 (8.86)	57 (5.96)	86 (9.22)	160 (8.26)	84 (4.33)	< 0.0001
Medication history for diabetes (*n*, %)	199 (2.46)	69 (2.97)	20 (2.09)	40 (4.29)	47 (2.43)	23 (1.19)	< 0.0001
Medication history for dyslipidemia (*n*, %)	168 (2.08)	56 (2.41)	17 (1.78)	26 (2.79)	39 (2.01)	30 (1.55)	< 0.0001

Data are presented as *n* (%)

Significant differences were found in all health-related behaviors and sociodemographic and clinical characteristics among the five commuting mode groups, with the exception of vegetable intake, excessive salt intake, and excessive sugar intake. Table 2 shows that physical activity frequency during leisure time was the lowest in the subway commuters followed by the car or taxi commuters, and physical activity intensity during leisure time was the lowest in the bus commuters followed by the subway commuters.

### Characteristics of the air pollutants

Summarized statistics of air pollutants for the study period in Beijing, China are presented in Additional file 1: Table S1. The means (standard deviations, SDs) of the air pollutants were 77.26 (4.63) μg/m^3^ for PM_2.5_, 107.78 (7.37) μg/m^3^ for PM10, 57.73 (3.46) μg/m^3^ for O_3_, 1.26 (0.07) mg/m^3^ for CO, 11.96 (0.91) μg/m^3^ for SO_2_, and 49.94 (2.70) μg/m^3^ for NO_2_.

### Association of commuting mode with dyslipidemia

**Fig. 2:**
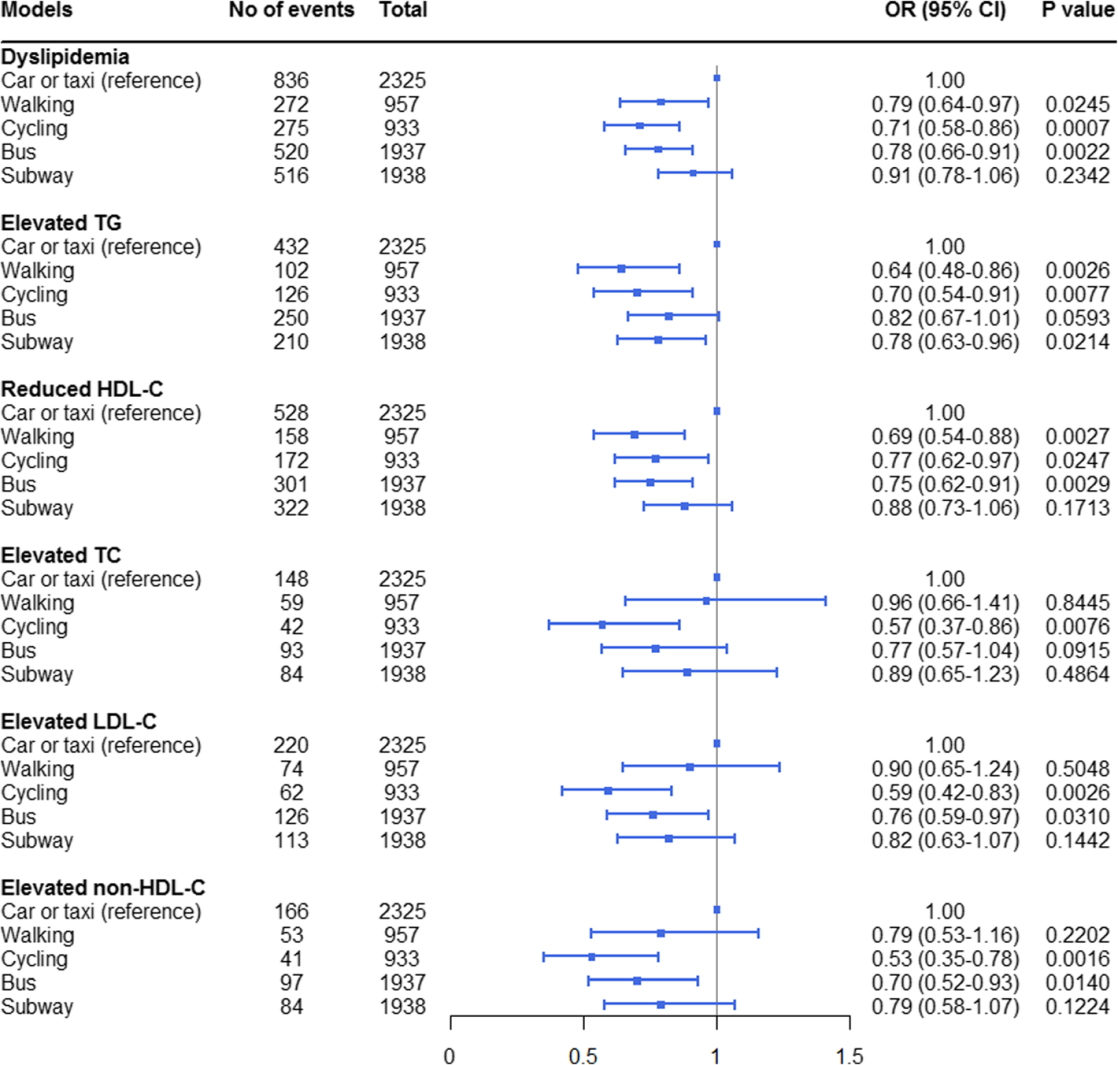
The association of commuting mode with dyslipidemia and its components. *TGs,* triglycerides; *HDL-C,* high-density lipoprotein cholesterol; *TC,* total cholesterol; *LDL-C,* low-density lipoprotein cholesterol; *non-HDL-C,* non-high-density lipoprotein cholesterol

The association between commuting mode and dyslipidemia was explored after adjusting for potential confounders (Fig. 2 and Additional file 1: Table S2).

Compared with travelling to work by car or taxi, travelling by walking (OR 0.80, 95% CI 0.66 to 0.99), cycling (OR 0.70, 95% CI 0.58 to 0.86) or taking the bus (OR 0.76, 95% CI 0.65 to 0.90) were all associated with a lower risk for dyslipidemia before adjusting for air pollutants. After adjusting for air pollutants, the association between the walking commuting mode (OR 0.79, 95% CI 0.64 to 0.97) and dyslipidemia was strengthened, and the associations between the cycling (OR 0.71, 95% CI 0.58 to 0.86) and taking the bus (OR 0.78, 95% CI 0.66 to 0.91) commuting modes and dyslipidemia were attenuated. However, adjusting for air pollutants did not alter the statistical significance of the associations (Fig. 2 and Additional file 1: Table S2). No significant association was found between subway commuting and dyslipidemia.

### Association of commuting mode with components of dyslipidemia

The associations of elevated TGs, reduced HDL-C, elevated TC, elevated LDL-C, and elevated non-HDL-C with commuting mode were also investigated (Fig. 2 and Additional file 1: Table S2).

Compared with the car or taxi commuting mode, the walking commuting mode was associated with a lower risk for elevated TGs and reduced HDL-C and the cycling commuting mode was associated with a lower risk for elevated TGs, reduced HDL-C, elevated TC, elevated LDL-C, and elevated non-HDL-C. Bus commuters had a lower risk for reduced HDL-C, elevated LDL-C and elevated non-HDL-C than car or taxi commuters. It was indicated that subway commuters had a lower risk for elevated TGs than car or taxi commuters. The adjustment for air pollutants also strengthened or attenuated the associations between commuting mode and the components of dyslipidemia, but the significance of the associations did not change.

### Dose-response trend of the association of dyslipidemia and its components with commuting mode by commuting time

**Fig. 3:**
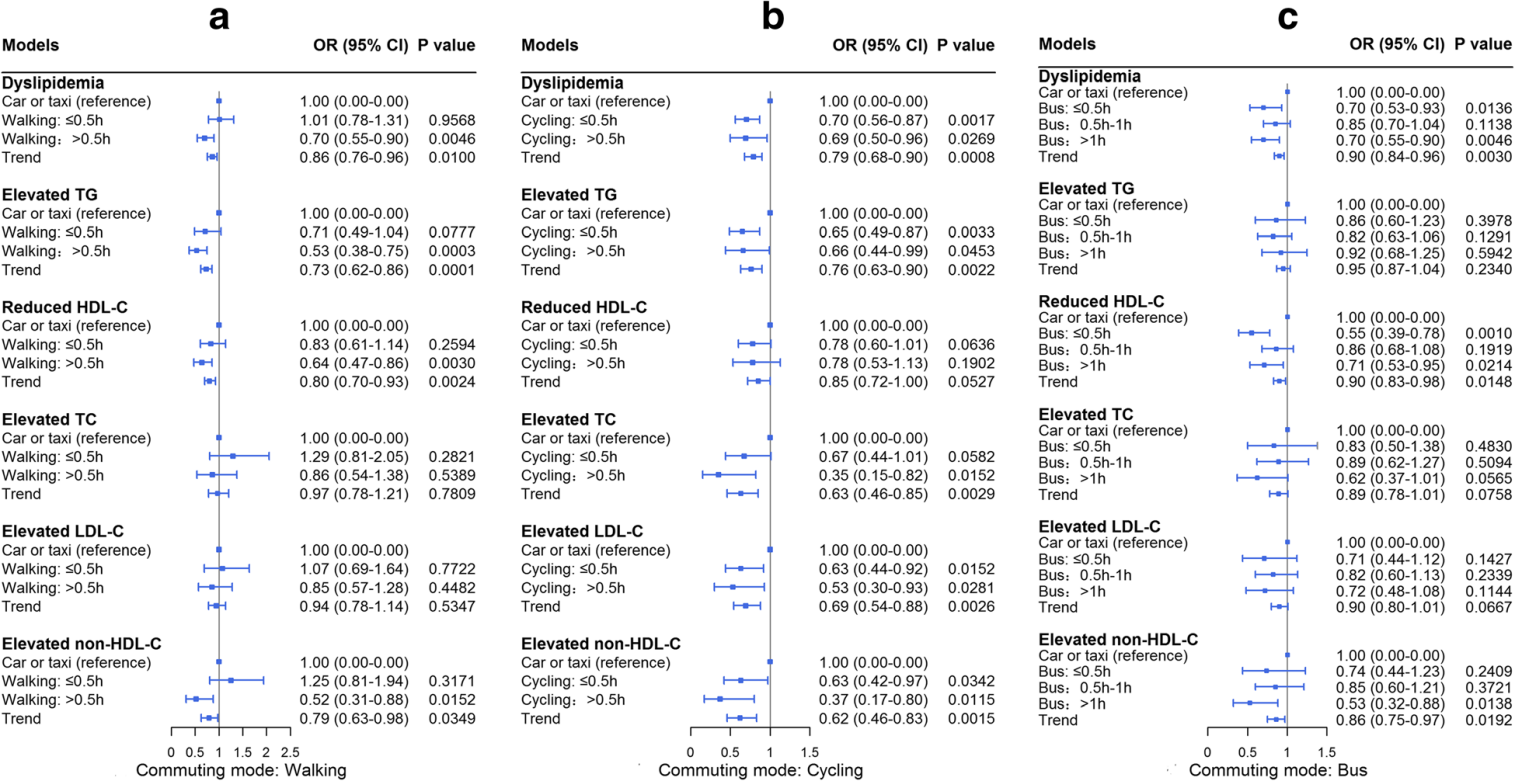
Dose-response trend of the association of dyslipidemia and its components with commuting mode by commuting time. *TGs,* triglycerides; *HDL-C,* high-density lipoprotein cholesterol; *TC,* total cholesterol; *LDL-C,* low-density lipoprotein cholesterol; *non-HDL-C,* non-high-density lipoprotein cholesterol

Figure 3 shows that among walking commuters, there were distinct dose-response trends for the prevalence of dyslipidemia, elevated TGs, reduced HDL-C, and elevated non-HDL-C by commuting time. Among cycling commuters, dose-response trends for the prevalence of dyslipidemia and all of its components were observed by commuting time. Among bus commuters, dose-response trends for the prevalence of dyslipidemia, reduced HDL-C, and elevated non-HDL-C were observed by commuting time.

## Discussion

To the best of our knowledge, this is the first study to investigate the association of dyslipidemia and its components (elevated TC, elevated TGs, reduced HDL-C, elevated LDL-C and elevated non-HDL-C) with commuting mode after taking air pollution into account. This study demonstrated the following: (1) the walking commuting mode was associated with a lower risk for dyslipidemia, elevated TGs and reduced HDL-C than the car or taxi commuting mode; (2) the cycling commuting mode was associated with a lower risk for dyslipidemia and each component of dyslipidemia than the car or taxi commuting mode; (3) bus commuters had a lower risk for dyslipidemia, reduced HDL-C, elevated LDL-C and elevated non-HDL-C than car or taxi commuters; (4) subway commuters had a lower risk for elevated TGs than car or taxi commuters; and (5) among walking, cycling, and bus-taking commuters, a dose-response trend of the association of dyslipidemia and some of its components with commuting mode by commuting time was observed.

The lower risks associated with travelling to work by walking, cycling, taking the bus or taking the subway for developing dyslipidemia and its components can be related to more overall daily physical activity. A negative association of bus and subway commuting with dyslipidemia and its components was also found in this study. People who travel to and from work by bus or subway may often stand rather than sit because there are many people on the bus or subway in the morning and evening peak hours in Beijing. The negative association of bus and subway commuting with dyslipidemia and its components may also be attributed to multimodal commuting, such as public transportation along with cycling or walking [39]. Further studies should consider evaluating not only the commuting mode but also the position during commuting (seated or standing) to verify the result. In this study population, people who travelled to and from work by car or taxi accounted for the largest percentage of the sample at 28.74% of the 8090 subjects. Compared with the data from 2006, the number of car or taxi commuters increased notably by more than three times (28.74% vs 9.96%), the number of people who commuted to work by cycling decreased from 39.06 to 11.53%, the percentage of walkers decreased from 21.97 to 11.83%, and the number of bus commuters decreased from 29.01 to 23.94% [9]. People who travel to work by car or taxi intend to save time in their commute. However, the road network in Beijing is becoming increasingly crowded because of the rapid rise in the number of car owners. Many problems arise as a result, such as traffic congestion, air pollution, frequent traffic accidents and other related issues. The traffic jams make these car or taxi commuters spend an increasing amount of time on their drive to and from work. A long time spent travelling by car or taxi may directly lead to obesity [39, 40], and obesity is the main cause of higher levels of serum lipids. Previous studies have assessed the association between dyslipidemia, elevated TGs, reduced HDL-C and commuting mode [9, 21]. One study in the adult population proved that the risk of dyslipidemia in workers who travel to work by bus, car or taxi is higher than that of workers who walk to work [9]. Active travel was negatively associated with TGs, but no significant association was found for commuting mode and HDL-C in the adult population [21]. The specific associations of elevated TC, elevated LDL-C, and elevated non-HDL-C with commuting mode have not been previously investigated.

The walking and cycling commuting modes are likely to increase physical activity, and thus, the risk for obesity and dyslipidemia is reduced for people who travel to work by walking or cycling. One previous study conducted in Latin America suggested that active travel, including walking or cycling, was negatively associated with metabolic syndrome, TGs and abdominal obesity [21]. No significant association between active commuting mode and low HDL-C was found in a previous study [21]. Another cross-sectional population-based study in a UK adult population showed that the walking and cycling commuting modes can reduce visceral adipose tissue and the development of cardiometabolic diseases, including dyslipidemia [39]. The study demonstrated that active commuting modes can help to reduce BMI and body fat percentage to a greater extent than passive commuting modes [39]. Cycling commuters tend to have greater overall physical activity and fitness than walking commuters. It was proven that cycling commuters have a lower risk for dyslipidemia and most of its components in this study. This finding may reflect the greater intensity of cycling compared with the intensity of walking in adults [41]. The results support the benefits of active commuting, particularly commuting by cycling. It was also found that people who commuted to work by walking or cycling with a longer commute had a lower risk for dyslipidemia than those who commuted for a shorter time in this study. This finding proved that to produce meaningful benefits for walking or cycling commuters, longer distances may be needed [15].

In previous studies, researchers have adjusted for many potential confounding factors, including dietary behavior [23, 24], physical activity [21–27, 30, 31] and some other confounding factors, but air pollutants were not considered. One field study of adults in Beijing investigated commuters’ exposure to air pollutants during their commute [42]. It has been reported that PM_2.5_ and CO exposure is greatly influenced by commuting mode. Furthermore, cyclists had higher exposures to PM_2.5_ and CO than bus or taxi commuters, and taxi commuters were exposed to lower concentrations of PM_2.5_ and higher concentrations of CO than bus commuters and cyclists [42]. The adverse effects of traffic-related air pollution on mortality or CVD have been well investigated in epidemiological studies [43, 44]. The air pollution level is high during morning or evening rush hours when many people are commuting. After adjusting for air pollutants, the association between walking commuting and dyslipidemia was strengthened, and the associations of cycling or taking the bus with dyslipidemia were attenuated. However, the significance of all the associations remained unchanged. When the air quality is very poor, people take protective measures, especially cycling and bus commuters with longer commuting times. The negative effect was attenuated after adjusting for air pollutants. However, the results need to be verified in large cohort studies.

### Strengths and limitations

This study has several strengths; it is the first study to investigate the association of dyslipidemia and its components with commuting mode after adjusting for air pollution. The second strength is that the associations between all components of dyslipidemia and commuting mode were assessed, thus generating more specific data for future studies. The third strength is that the dose-response trend of the association of dyslipidemia and its components with commuting mode by commuting time was investigated. The fourth strength is that the study indicated that the walking or cycling commuting modes should be encouraged, especially for people with a higher risk of dyslipidemia who usually go to work by car or taxi.

Several limitations should be considered in the present study. The first limitation is that the study is cross-sectional, which means that the causal relationships between commuting mode and dyslipidemia cannot be established. Prospective cohort studies should be conducted in the future to verify the results. The second limitation is that the questionnaire was not validated and was self-reported, and the measures may underestimate or overestimate the results. The third limitation is that the air pollutants’ values are at the district level and not at the personal level. In future studies, we will measure the personal level of air pollutants to generate a more accurate association of commuting mode with dyslipidemia.

## Conclusions

This study revealed that the walking, cycling and bus-taking commuting modes can reduce the risk of dyslipidemia and some of its components. Prevention education should be emphasized among higher-risk people who usually go to work by car or taxi. Population health may be improved by policies that increase active commuting, particularly walking or cycling with longer commutes.

## Additional file

Additional file 1: **Table S1.** Summary statistics of air pollutants in urban areas in Beijing during the study period. **Table S2.** Risk of commuting mode for dyslipidemia, elevated TGs, reduced HDL-C, elevated TC, elevated LDL and non-HDL-C. (DOCX 18 kb)

## Abbreviations

BMI: Body mass index
CI: Confidence intervals
CVDs: Cardiovascular diseases
DBP: Diastolic blood pressure
FPG: Fasting plasma glucose
HDL-C: High-density lipoprotein cholesterol
LDL-C: Low-density lipoprotein cholesterol
MAP: Mean arterial pressure
SBP: Systolic blood pressure
TC: Total cholesterol
TGs: Triglycerides
