# Supplementary material for: Association of commuting mode with dyslipidemia and its components after accounting for air pollution in the working population of Beijing, China
Source: BMC Public Health. 2019 May 22;19:622. doi: 10.1186/s12889-019-6887-x (PMC6530144; doi:10.1186/s12889-019-6887-x)
Supplement: Supplementary file 1 — Table S1. Summary statistics of air pollutants in urban areas in Beijing during the study period. Table S2. Risk of commuting mode for dyslipidemia, elevated TGs, reduced HDL-C, elevated TC, elevated LDL and non-HDL-C. (DOCX 18 kb) [file 12889_2019_6887_MOESM1_ESM.docx]

**Table S1** Summary statistics of air pollutants in urban areas in Beijing during the study period

| Variables | Mean | SD | Percentiles | | | | |
| --- | --- | --- | --- | --- | --- | --- | --- |
|  |  |  | Min | P_25_ | P_50_ | P_75_ | Max |
| PM_2.5_ (μg/m^3^) | 77.26 | 4.63 | 69.12 | 72.59 | 77.96 | 79.06 | 90.08 |
| PM_10_ (μg/m^3^) | 107.78 | 7.37 | 96.30 | 99.15 | 109.29 | 110.31 | 124.19 |
| O_3_ (μg/m^3^) | 57.73 | 3.46 | 52.33 | 55.06 | 57.99 | 59.92 | 75.77 |
| CO (mg/m^3^) | 1.26 | 0.07 | 1.18 | 1.22 | 1.25 | 1.31 | 1.49 |
| SO_2_ (μg/m^3^) | 11.96 | 0.91 | 10.61 | 11.39 | 11.84 | 12.10 | 15.22 |
| NO_2_ (μg/m^3^) | 49.94 | 2.70 | 29.61 | 48.87 | 49.74 | 50.74 | 59.88 |

SD: standard deviation

**Table S2** Risk of commuting mode for dyslipidemia, elevated TG, reduced HDL-C, elevated TC, elevated LDL and non-HDL-C

|  | Model | Car or taxi | Walking | Cycling | Bus | Subway |
| --- | --- | --- | --- | --- | --- | --- |
| Dyslipidemia | Model 1 | Reference | 0.81 (0.68-0.96) | 0.69 (0.59-0.82) | 0.73 (0.64-0.84) | 0.81 (0.70-0.93) |
|  | Model 2 | Reference | 0.80 (0.66-0.99) | 0.70 (0.58-0.86) | 0.76 (0.65-0.90) | 0.89 (0.76-1.05) |
|  | Model 3 | Reference | 0.79 (0.64-0.97) | 0.71 (0.58-0.86) | 0.78 (0.66-0.91) | 0.91 (0.78-1.06) |
| Elevated TG | Model 1 | Reference | 0.60 (0.48-0.77) | 0.64 (0.51-0.79) | 0.74 (0.62-0.88) | 0.71 (0.59-0.85) |
|  | Model 2 | Reference | 0.65 (0.49-0.87) | 0.69 (0.54-0.90) | 0.81 (0.66-0.99) | 0.77 (0.62-0.96) |
|  | Model 3 | Reference | 0.64 (0.48-0.86) | 0.70 (0.54-0.91) | 0.82 (0.67-1.01) | 0.78 (0.63-0.96) |
| Reduced HDL-C | Model 1 | Reference | 0.72 (0.59-0.88) | 0.73 (0.60-0.89) | 0.69 (0.59-0.82) | 0.75 (0.64-0.88) |
|  | Model 2 | Reference | 0.71 (0.55-0.90) | 0.76 (0.61-0.96) | 0.73 (0.61-0.88) | 0.85 (0.71-1.02) |
|  | Model 3 | Reference | 0.69 (0.54-0.88) | 0.77 (0.62-0.97) | 0.75 (0.62-0.91) | 0.88 (0.73-1.06) |
| Elevated TC | Model 1 | Reference | 0.97 (0.71-1.32) | 0.69 (0.49-0.99) | 0.74 (0.57-0.97) | 0.67 (0.51-0.88) |
|  | Model 2 | Reference | 0.95 (0.65-1.39) | 0.59 (0.39-0.89) | 0.79 (0.59-1.07) | 0.91 (0.66-1.25) |
|  | Model 3 | Reference | 0.96 (0.66-1.41) | 0.57 (0.37-0.86) | 0.77 (0.57-1.04) | 0.89 (0.65-1.23) |
| Elevated LDL-C | Model 1 | Reference | 0.80 (0.61-1.06) | 0.68 (0.51-0.91) | 0.67 (0.53-0.84) | 0.59 (0.47-0.75) |
|  | Model 2 | Reference | 0.90 (0.65-1.24) | 0.59 (0.42-0.83) | 0.76 (0.59-0.97) | 0.82 (0.63-1.07) |
|  | Model 3 | Reference | 0.90 (0.65-1.24) | 0.59 (0.42-0.83) | 0.76 (0.59-0.97) | 0.82 (0.63-1.07) |
| Elevated non-HDL-C | Model 1 | Reference | 0.87 (0.63-1.20) | 0.56 (0.40-0.80) | 0.73 (0.57-0.95) | 0.81 (0.61-1.06) |
|  | Model 2 | Reference | 0.78 (0.53-1.14) | 0.53 (0.36-0.79) | 0.71 (0.53-0.94) | 0.79 (0.58-1.06) |
|  | Model 3 | Reference | 0.79 (0.53-1.16) | 0.53 (0.35-0.78) | 0.70 (0.52-0.93) | 0.79 (0.58-1.07) |

Abbreviations: *TG,* triglycerides; *HDL-C,* high-density lipoprotein cholesterol; *TC,* total cholesterol; *LDL-C,* low-density lipoprotein cholesterol; *non-HDL-C,* non high-density lipoprotein cholesterol.

Model 1: adjusted for age and gender

Model 2: adjusted for all covariates in Model 1, as well as body mass index, education level, commuting time, self–reported work pressure, physical activity frequency, physical activity intensity, sleep duration, smoking status, drinking status, a mainly vegetable diet, excessive meat intake, excessive fat intake, excessive salt intake, excessive sugar intake, medication history for hypertension, diabetes, and dyslipidemia, mean arterial pressure and fasting plasma glucose.

Model 3: adjusted for all covariates in Model 2, as well as PM_25_, PM_10_, NO_2_, SO_2_, CO and O_3_
